# 1,2-Substituted 4-(1*H*)-Quinolones: Synthesis, Antimalarial and Antitrypanosomal Activities *in Vitro*

**DOI:** 10.3390/molecules190914204

**Published:** 2014-09-10

**Authors:** Abraham Wube, Antje Hüfner, Werner Seebacher, Marcel Kaiser, Reto Brun, Rudolf Bauer, Franz Bucar

**Affiliations:** 1 Department of Pharmacognosy, Institute of Pharmaceutical Sciences, University of Graz, Universitätsplatz 4/1, A-8010 Graz, Austria; E-Mails: abraham.wube@uni-graz.at (A.W.); rudolf.bauer@uni-graz.at (R.B.); 2 Department of Pharmaceutical Chemistry, Institute of Pharmaceutical Sciences, University of Graz, Universitätsplatz 1, A-8010 Graz, Austria; E-Mails: antje.huefner@uni-graz.at (A.H.); we.seebacher@uni-graz.at (W.S.); 3 Swiss Tropical and Public Health Institute, Socinstrasse 57, CH-4002 Basel, Switzerland; 4 University of Basel, Petersplatz 1, CH-4003 Basel, Switzerland; E-Mails: Marcel.Kaiser@unibas.ch (M.K.); reto.brun@unibas.ch (R.B.)

**Keywords:** 4-(1*H*)-quinolone, antimalarial, antitrypanosomal, cytotoxicity, SAR

## Abstract

A diverse array of 4-(1*H*)-quinolone derivatives bearing substituents at positions 1 and 2 were synthesized and evaluated for antiprotozoal activities against *Plasmodium falciparum* and *Trypanosoma brucei rhodesiense*, and cytotoxicity against L-6 cells *in vitro*. Furthermore, selectivity indices were also determined for both parasites. All compounds tested showed antimalarial activity at low micromolar concentrations, with varied degrees of selectivity against L-6 cells. Compound **5a** was found to be the most active against *P. falciparum*, with an IC_50_ value of 90 nM and good selectivity for the malarial parasite compared to the L-6 cells. Compound **10a**, on the other hand, showed a strong antitrypanosomal effect with an IC_50_ value of 1.25 µM. In this study side chain diversity was explored by varying the side chain length and substitution pattern on the aliphatic group at position-2 and a structure-antiprotozoal activity study revealed that the aromatic ring introduced at C-2 contributed significantly to the antiprotozoal activities.

## 1. Introduction

According to the latest World Health Organization report there were an estimated 207 million cases of malaria in 2012 and an estimated 627,000 deaths [1]. Sub-Saharan Africa is the region most affected by the malaria epidemic, accounting for 90% of all malaria mortality, 77% if which occurs in children under the age of five. Beyond the human toll, malaria has significant economic impacts in endemic countries, costing sub-Saharan Africa an estimated $12 billion in lost GDP every year and consuming 40% of all public health spending [2]. As a result, the burden of the disease on societies and economies is tremendous.

*Trypanosoma brucei rhodesiense* causes sleeping sickness in East and South Africa, while *T. b. gambiense* causes sleeping sickness in Central and West Africa. Both forms of the disease affect mainly poor rural people who have poor access to basic health facilities in their vicinity. Between 1900 and 1915 the disease killed more than a quarter of a million people in Africa [3]. The drugs currently available to treat the advanced stage of sleeping sickness are the arsenical drug melarsoprol used for *T. b. rhodesiense*, and the nifurtimox-eflornithine combination therapy (NECT) used for *T. b. gambiense*. Melarsoprol is highly toxic and shows efficacy problems, while NECT is difficult to apply. Two new molecules, fexinidazole and the oxaborole SCYX-7158, are in clinical trials, but it is uncertain if and when they will make it to the market [4].

Quinolones, which were evolved from agents primarily used for the treatment of urinary tract infections, have been used extensively for the treatment of a broad range of clinical infections such as gastrointestinal, respiratory tracts, as well as infections of the bone, joints and skin. Although a wide variety of quinolones, mainly fluoroquinolones, had been intensively studied, quinolones possessing aliphatic substituents at position 2, but devoid of a carboxyl or other substituents at position 3, have not been investigated intensively. The antimalarial property of endochin, which possesses a quinolone pharmacophore nucleus, and its ester derivatives were first reported in the mid-1900 [5]. A recently renewed interest on the antiplasmodial activities of endochin derivatives highlighted 4-(1*H*)-quinolones as promising antimalarial agents [6,7,8,9,10]. Interestingly, Pidathala *et al.* for example, have identified 2-bisaryl-3-methyl-4-(1*H*)-quinolones as inhibitors of the *P. falciparum* oxidoreductase (PfNDH2) involved in the redox reaction of NADH oxidation with subsequent quinol production [11]. The 4-(1*H*)-quinolone derivatives investigated so far contain a bromine, a carboxyl, a carboxyl ester, an alkyl, an alkynyl and phenyl groups at C-2; and a hydroxyl group at position-1 or no substituents at position-1. Our new series of compounds contains unique substituents including alkyls, alkenyl, alkynyl, and 1-bromoethyl at position-1; aliphatic groups having various degrees and position of unsaturation with and without aryl groups, and no substituents at C-3. 4-(1*H*)-Quinolone derivatives including the well-known antibiotic fluoroquinolones such as ciprofloxacin, gatifloxacin and moxifloxacin display potential antitrypanosomal effects [12]. This is further supported by a recent report by Hiltonsperger *et al.* that 4-(1*H*)-quinolones with a benzyl amide group in position 3 and cyclic or acyclic amines in position 7 exhibited high antitrypanosomal activity [13]. Thus, 4-(1*H*)-quinolones represent a new sub-class of promising antitrypanosomal agents for further development. Hence, herein we report the synthesis of a series of 1,2-substituted-4(1*H*)-quinolones, and examine their antiprotozoal and cytotoxic properties *in vitro*.

## 2. Results and Discussion

### 2.1. Synthesis

**Scheme 1:**
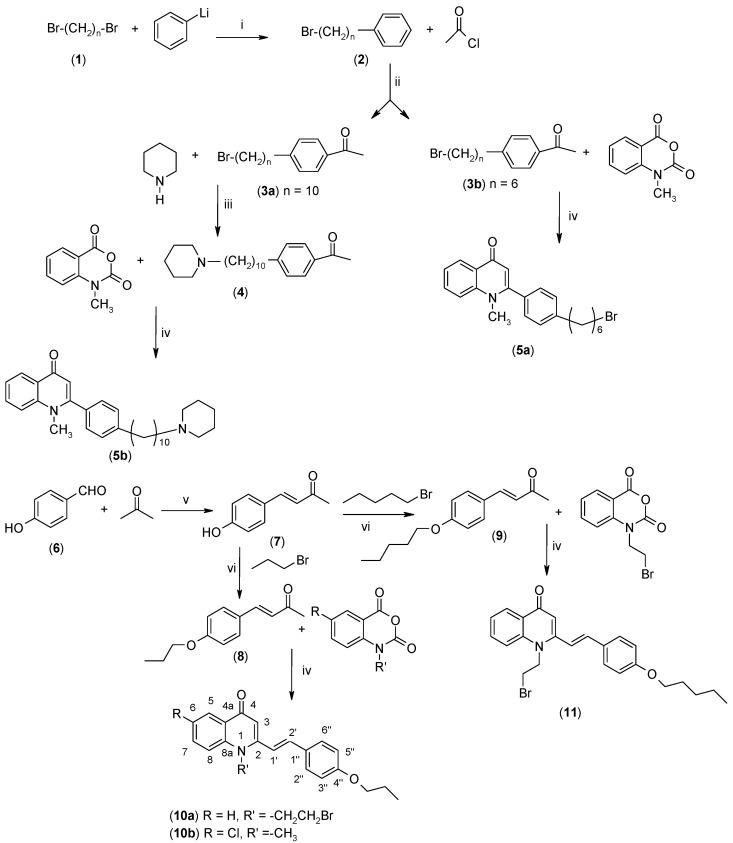
Synthesis of 4-(1*H*)-quinolone derivatives with phenyl-bearing aliphatic substituent at C-2.

As illustrated in Scheme 1, the methyl ketones **3a**–**b** were prepared from ω-bromoalkylbenzene via Friedel-Crafts-acetylation, which was initiated by the reaction between α,ω-dibromoalkane (**1**) and phenyl lithium.

*Reagents and conditions*: (i) THF, 0 °C; (ii) AlCl_3_, CH_2_Cl_2_, 0 °C; (iii) K_2_CO_3_, DMF, RT; (iv) LDA, THF, −78 °C; (v) 2.0 N NaOH, RT; (vi) K_2_CO_3_, acetone, reflux.

**Scheme 2:**
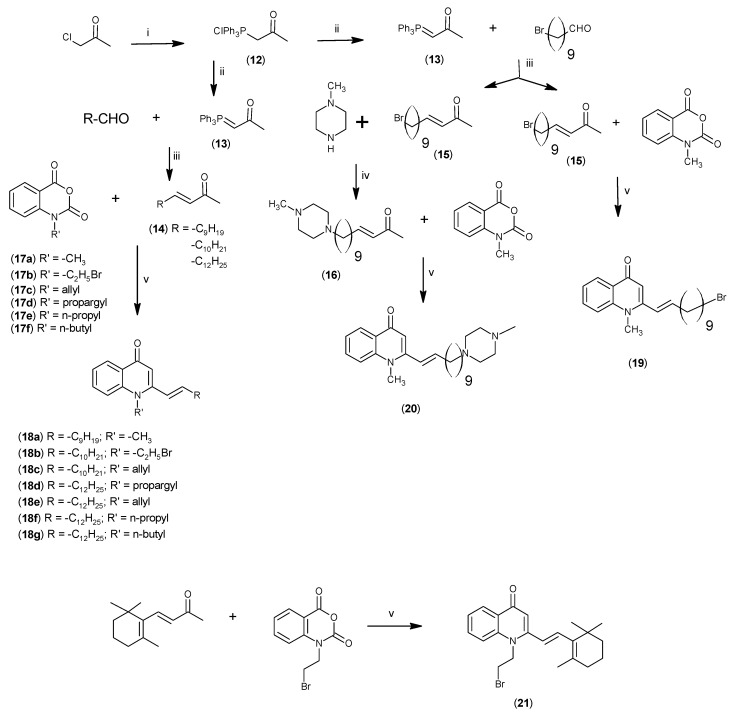
Synthesis of 4-(1*H*)-quinolones with (*E*)-α,β-alkenyl substituents.

Substitution of the bromine of **3a** with piperidine afforded **4**. The (*E*)-4-(4-alkoxyphenyl)-3-buten-2-ones **8**, **9**, on the other hand, were generated from 4-hydroxybenzaldehyde (**6**) and acetone by the action of a strong base and further condensation with 1-bromo-alkane. The methyl-α,β-(*E*)-alkenyl ketones **15** and **16**, which served as important intermediates for the synthesis of our diverse array of 4-(1*H*)-quinolone derivatives (Scheme 2) were obtained using our previously reported procedure [14].

*Reagents and conditions*: (i) PPh_3_, AcCN, reflux; (ii) 1 N NaOH, pH 7–8; (iii) THF, reflux, 24 h; (iv) K_2_CO_3_, DMF, RT; (v) LDA, THF, −78 °C.

**Scheme 3:**
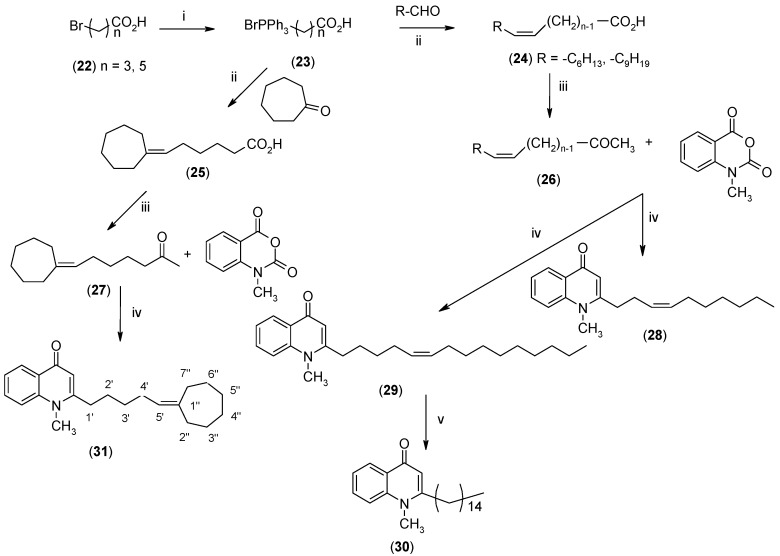
Synthesis of (*Z*)-alkenyl-bearing 4-(1*H*)-quinolones.

The commercially available chloroacetone was first treated with triphenylphosphine to give **12**, followed by alkaline elimination to afford the ylide **13**. Wittig olefination of **13** with the corresponding aldehydes gave rise to the methyl-(*E*)-alkenyl ketones **14**–**15** and subsequent introduction of 1-methyl-piperazine to the ω-bromo-α,β-(*E*)-alkenyl ketones **15** gave **16**. The *Z*-olefin-bearing methyl ketones **26** and **27**, which appear in Scheme 3, were prepared by Wittig condensation of either aldehydes or cycloheptanone with bromotriphenylphosphincarboxylic acid **23**, which was obtained by treatment of ω-bromocarboxylic acid **22** with triphenylphosphine.

*Reagents and conditions*: (i) PPh_3_, AcCN, reflux; (ii) NaN(SiMe_2_)_3_, THF; (iii) MeLi, THF, 0 °C; (iv) LDA, THF, −78 °C; (v) H_2_, 10% Pd/C, C_2_H_5_OH.

**Scheme 4:**
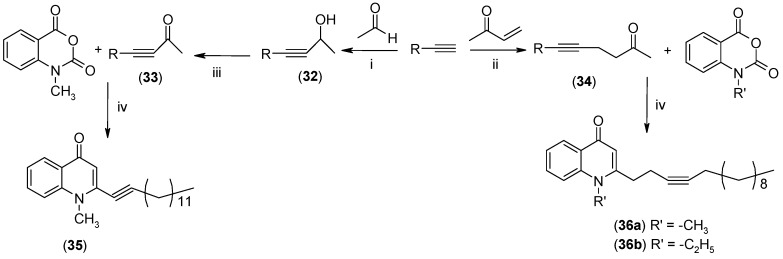
Synthesis of alkynyl-bearing 4-(1*H*)-quinolones.

In order to assess the importance of degree of unsaturation on the antiprotozoal activity, triple bond-bearing methyl ketones were also synthesized. 1-Alkynes were converted to α,β- and γ,δ-alkynones as shown in Scheme 4. Treatment of 1-undecyne and methyl vinyl ketone with palladium (II) acetate provided γ,δ-hexadecy-2-one **34**. The α,β-hexadecynone **33**, on the other hand, was generated by the reaction between 1-tridecyne and acetaldehyde, followed by oxidation with manganese dioxide.

*Reagents and conditions*: (i) n-BuLi, THF, −78 °C; (ii) Pd(OAc)_2_, PMe_3_, acetone, 60 °C; (iii) MnO_2_, THF; (iv) LDA, THF, −78 °C.

The isatoic anhydride derivatives bearing ethyl, *n*-propyl, *n*-butyl, allyl, propargyl and 2-bromoethyl, which served as important intermediates for the synthesis of our 4-(1*H*)-quinolone derivatives were prepared by treatment of the commercially available isatoic anhydride with the corresponding halolalkanes, allyl bromide, propargyl bromide and 1,2-dibromoethane by a previously described method [15].

Finally, treatment of the methyl ketones with isatoic anhydride derivatives in the presence of LDA afforded the desired 4-(1*H*)-quinolones **5a**–**b**, **10a**–**b**, **11** (Scheme 1), **18a**–**g**, **19**–**21** (Scheme 2), **28**–**31** (Scheme 3), **35** and **36**–**b** (Scheme 4). 1-(2-Bromoethyl)-2-[(1′*E*)-2′-(2′′,6′′,6′′-trimethyl-1′′-cyclo-hexen-1′′-yl)ethenyl]-4-(1*H*)-quinolone (**21**) was synthesized from the commercially available ketone, β-ionone.

### 2.2. Biological Evaluations

The antimalarial property of our 4-(1*H*)-quinolone derivatives was determined *in vitro* against the chloroquine sensitive strain of *P. falciparum* NF54 and their IC_50_ values are listed in Table 1. The compounds displayed moderate to strong antimalarial effect at micromolar and submicromolar concentrations. Among the set of compounds bearing α,β-(*E*)-alkenyls at position 2, compounds **18e** having an allyl at position 1 and tetradecenyl at C-2 showed superior inhibitory effect (IC_50_ value of 0.31 µM), whereas compound **18a** bearing a methyl and undecenyl groups at positions 1 and 2, respectively, was found to be the least potent (IC_50_ value of 12.4 µM). Comparison of the activities of compound **18d**–**g** having the same aliphatic groups at C-2 but differing in the substituents at position 1, a substantial variation in activities was recognized, indicating that the allyl group in compound **18e** contributed significantly to the antimalarial activity. Compound **18d** bearing a propargyl group at position 1 displayed four-fold and ten-fold less potency than compounds **18f** and **18e**, bearing n-propyl and allyl groups, respectively. Introduction of a bromine atom at the end of the aliphatic group at C-2 markedly enhanced antimalarial potency (*cf.* compounds **19** and **18a**). However, replacement of the bromine of compound **19** by 1-methylpiperazine in compound **20** results in an almost two-fold loss in potency.

Of the compounds containing (*Z*)-alkenyl moieties (compounds **28** and **29**), compound **29** showed superior potency and better selectivity to *P. falciparum*, indicating that antimalarial potency increases with increases in lipophilicity. However, hydrogenation of the alkenyl chain of **29** to afford **30** gave rise to a 16-fold loss in potency compared to **29**, but this compound is still more active than compound **28** having a decenyl group at position 2, suggesting that unsaturation is a key to retaining potency. It is noteworthy that among the three alkynyl bearing compounds **36b** was the most potent against the malarial parasite (IC_50_ = 0.45 µM), where **36b** was two-fold more potent than **35** and seven-fold more potent than **36a**. Similarly, the alkynyl bearing 4-(1*H*)-quinolones, **35** and **36a**–**b** displayed impressive inhibitory effects compared to the corresponding alkenyl containing analogs. Thus, it’s reasonable to suggest that unsaturation and lipophilicity are essential structural features for improved antimalarial potency.

**Table 1:** Antimalarial, antitrypanosomal and cytotoxic activities of 4-(1*H*)-quinolone derivatives.

Comp.	Structure	IC_50_ (µM)			SI	
		NF54	STIB900	L-6	L-6/NF54	L-6/STIB900
5a	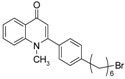	0.09	1.64	6.63	73.33	4.04
5b	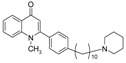	0.82	3.41	9.63	11.73	2.83
10a	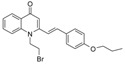	1.58	1.25	25.83	16.45	20.58
10b	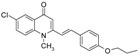	0.47	21.89	161.2	342.89	7.36
11	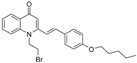	1.56	3.82	42.39	27.11	11.10
18a	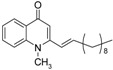	12.40	52.22	44.72	3.61	0.86
18b	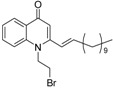	2.30	8.28	38.35	16.69	4.63
18c	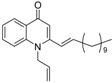	2.90	21.23	19.82	6.82	0.93
18d	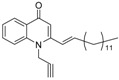	4.64	24.48	24.68	5.32	1.01
18e	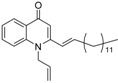	0.31	8.18	14.01	45.78	1.71
18f	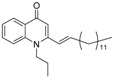	1.19	19.08	13.04	10.99	0.68
18g	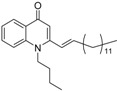 >	2.29	18.73	13.26	5.79	0.70
19	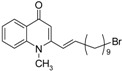	0.69	1.07	36.31	52.64	33.93
20	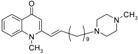	1.35	4.94	32.54	24.14	6.59
21	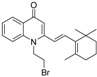	1.99	8.85	33.25	16.65	3.76
28	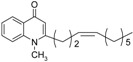	15.30	11.04	49.46	3.22	4.48
29	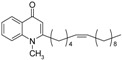	0.39	10.82	13.15	33.84	1.16
30	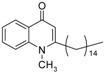	6.40	42.41	101.3	15.91	2.39
31	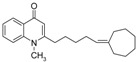	10.11	14.37	45.80	4.52	3.19
35	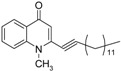	0.84	10.51	15.80	18.78	1.50
36a	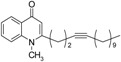	3.11	33.48	13.3	4.29	0.39
36b	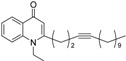	0.45	16.60	13.56	29.82	0.82
Chloroquine		0.0063				
Melarsoprol			0.0075			
Podophyllotoxin				0.017		

As shown in Table 1, introduction of a phenyl group in the aliphatic side chain at C-2 dramatically increased the antimalarial potency. Compounds **5a**–**b**, **10a**–**b** and **11** were found to be the most potent with IC_50_ values ranging from 90 nM to 1.58 µM. Interestingly, compound **5a** having a phenyl group α to the quinolone nucleus and a bromine atom at the end of the aliphatic side chain appears to be the most potent antimalarial agent (IC_50_ value of 0.09 µM). Like compound **19**, replacement of the bromine with a *sec*-cycloamino group decreases the antimalarial activity. Therefore, a bromine atom at the end of the aliphatic side chain is detrimental for antimalarial activity. Our finding supports the notion that aromatic groups at position 2 of the quinolone nucleus could potentially enhance antimalarial activities [11]. Comparison of the antimalarial vs cytotoxicity profile of compounds **10a** and **10b**, the four-fold increase in antimalarial activity of compound **10b** is accompanied with marked increase in selectivity. Thus, introduction of chlorine at C-6 of compound **10b** drastically improved the selectivity to the malarial parasite compared to the rat skeletal muscle cell. Except, compounds **18a**, **18c**, **18d**, **18g**, **28**, **31** and **36a**, the remaining 4-(1*H*)-quinolone derivatives displayed moderate to good selectivity with selectivity indices values >10.0.

Our 4-(1*H*)-quinolone derivatives were further assessed for their antitrypanosomal property against *T. b. rhodesiense in vitro* and the 50% inhibitory concentration and selectivity indices are calculated as the quotient of cytotoxicity and antitrypanosomal IC_50_ values. As shown in Table 1, the compounds displayed weak inhibitory effects. Nevertheless, it is worth mentioning that the best activity was displayed by compounds **10a** and **19**, with IC_50_ values of 1.25 and 1.07 µM, respectively. Moreover, these compounds were found to be most selective for the trypanosomal parasite with selectivity index values of 20.6 and 34.0, respectively.

## 3. Experimental

### 3.1. General Information

The drug sensitive strain of *P. falciparum* (NF54) was obtained from Schipol Airport, The Netherlands [16]. The stock of *T. b. rhodesiens*e was isolated in 1982 from a human patient in Tanzania and after several mouse passages cloned and adapted to axenic culture conditions [17]. L-6 cells were derived from rat skeletal myoblasts [18,19].

All chemicals were purchased from Sigma-Aldrich (Munich, Germany). Reactions were carried our using oven-dried glassware under an atmosphere of argon. DMF was distilled from CaH_2_. THF was distilled from sodium and stored over molecular sieve (4 Å). IR spectra obtained on a Bruker ALPHA Platinum ATR A220/D-OX (Bruker, Billerica, MA, USA) unless otherwise stated. 1H and 13C-NMR spectra were recorded at 400 and 100 MHz, respectively on a Varian 400 MHz spectrometer (Varian, Palo Alto, CA, USA) using deuterated chloroform as solvent with TMS as internal standard. Mass Spectra were obtained by LC-ESI-MS analysis in positive mode on an UltiMate 3000 RS HPLC system coupled to a LTQ XL mass spectrometer (Thermo Scientific, West Palm Beach, FL, USA). Precoated Si gel 60 F254 Plates (Merck, Darmstadt, Germany) were used to monitor the progress of the reactions and column fractions. Spots were detected by UV/254 nm and spraying with molybdatophosphoric acid and subsequent heating. Compounds were purified by column chromatography on Si gel 60 (0.063–0.200 mm) using cyclohexane/ethyl acetate mixtures as eluent.

### 3.2. Synthesis

#### 3.2.1. Synthesis of 4-(ω-Bromoalkyl)acetophenone (**3**)

To a stirred solution of α,ω-dibromoalkane (2.5 equiv.) in dry THF cooled to 0 °C, phenyl lithium (1 equiv.) was added drop wise in an inert atmosphere. The reaction mixture was first stirred for 2 h at 0 °C and then for 10 h at room temperature. Ice was added to quench the reaction, followed by extraction with dichloromethane, and the organic extract was dried with Na_2_SO_4_. The concentrated crude product was purified by silica gel column chromatography (CC) eluting with cyclohexane/ethyl acetate (95:5) to afford ω-bromoalkylbenzene (**2**) [20]. To a stirred mixture of AlCl_3_ (1 equiv.) and acetyl chloride (0.5 equiv.) in dry dichloromethane at 0 °C, a solution of ω-bromoalkylbenzene (**2**) (1 equiv.) in acetyl chloride (0.5 equiv.) was added. After 3 h of further stirring at 0 °C, the mixture was poured into a mixture of conc. HCl (~15 mL) and ice (150 g), the aqueous layer was extracted with dichloromethane, the combined organic phase was washed with 1.0 N NaOH, water and brine; dried and concentrated. Purification of the crude product by CC eluting with cyclohexane/ethyl acetate (9:1) yielded compounds **3a**–**b** as yellow oils (82%–87%).

*1-(4-(10-Bromodecyl)phenyl)ethanone* (**3a**) was prepared from 10-bromodecylbenzene (5.0 g, 16.8 mmol), AlCl_3_ (2.20 g, 16.8 mmol), acetyl chloride (1.32 g, 16.8 mmol) in CH_2_Cl_2_ (25 mL) as a yellow oil (82.6%). ^1^H-NMR δ: 7.87 (d, *J* = 8.0 Hz, 2H), 7.25 (d, *J* = 8.0 Hz, 2H), 3. 39 (t, *J* = 6.8 Hz, 2H), 2.65 (t, *J* = 7.6 Hz, 2H), 2.57 (s, 3H), 1.84 (quint, *J* = 6.8 Hz, 2H), 1.62 (quint, *J* = 6.8 Hz, 2H), 1.42–1.37 (m, 4H), 1.30–1.27 (m, 10H). ^13^C-NMR δ: 197.7, 148.7, 134.8, 128.7, 128.5, 35.9, 35.7, 33.9, 32.7, 31.0, 30.1, 29.3, 29.1, 28.0, 26.6, 26.3.

*1-(4-(6-Bromohexyl)phenyl)ethanone* (**3b**) was prepared from 6-bromohexylbenzene (5.0 g, 20.8 mmol), AlCl_3_ (2.73 g, 20.8 mmol), acetyl chloride (1.64 g, 20.8 mmol) in CH_2_Cl_2_ (25 mL) as a yellow oil (86.8%). ^1^H-NMR δ: 7.87 (d, *J* = 8.0 Hz, 2H), 7.25 (d, *J* = 8.0 Hz, 2H), 3. 38 (t, *J* = 7.6 Hz, 2H), 2.66 (t, *J* = 7.6 Hz, 2H), 2.57 (s, 3H), 1.83 (quint, *J* = 6.4 Hz, 2H), 1.64 (quint, *J* = 6.8 Hz, 2H), 1.46 (quint, *J* = 6.8 Hz, 2H), 1.35 (quint, *J* = 6.8 Hz, 2H). ^13^C-NMR δ: 197.6, 148.2, 134.8, 128.7, 128.4, 35.6, 35.4, 33.7, 32.5, 30.7, 26.6, 26.2.

#### 3.2.2. Synthesis of 1-(4-(10-Piperidinyldecyl)phenyl)ethanone (**4**)

Anhydrous K_2_CO_3_ (3.26 g, 23.6 mmol, 4.0 equiv.) was added to a solution of piperidine (0.75 g, 8.9 mmol, 1.5 equiv.) in dry DMF (20 mL) in argon and the mixture was stirred for 45 min. 1-(4-(10-bromodecyl)phenyl)ethanone (**3a**) (2.0 g, 5.9 mmol, 1 equiv.) dissolved in DMF (10 mL) was added and stirring was continued for 24 h at room temperature. The reaction mixture was diluted with water (20 mL) and extracted with dichloromethane, washed with water several times and brine, dried over Na_2_SO_4_, concentrated to give 2.5 g of crude product. CC purification afforded 1.8 g of a light yellow oil (88.9%). ^1^H-NMR δ: 7.87 (d, *J* = 8.0 Hz, 2H), 7.25 (d, *J* = 8.0 Hz, 2H), 2.65 (t, *J* = 7.6 Hz, 2H), 2.58 (s, 3H), 2.36 (bs, 4H), 2.26 (t, *J* = 7.6 Hz, 2H), 1.64–1.55 (m, 6H), 1.47 (quint, *J* = 6.8 Hz, 2H), 1.43 (quint, *J* = 6.8 Hz, 2H), 1.31–1.22 (m, 12H). ^13^C-NMR δ: 197.7, 148.7, 134.8, 128.5, 128.3, 59.6, 54.5, 35.9, 31.0, 29.5, 29.4, 29.4, 29.3, 29.1, 29.0, 27.5, 26.8, 25.9, 24.4.

#### 3.2.3. Synthesis of (*E*)-4-(4-Hydroxyphenyl)-3-buten-2-one (**7**)

To a mixture of 4-hydroxybenzaldehyde (10.0 g, 81.9 mmol, 1 equiv.) and acetone (19.0 g, 328 mmol, 4 equiv.) 2.0 N NaOH solution (20 mL) was added and the mixture stirred in an ice/H_2_O bath for 12 h. After acidifying with 1.0 N HCl solution the excess acetone was evaporated and the residue was extracted with ethyl acetate, washed with water and dried to yield a yellowish crude product. CC purification with cyclohexane/ethyl acetate (9:1) gave a yellow solid (11.0 g) (83%) [21].

#### 3.2.4. Synthesis of (*E*)-4-(4-Alkoxyphenyl)-3-buten-2-ones **8**–**9**

A mixture of **7** (1.0 equiv.) in acetone (25 mL), 1-bromoalkane (1.2 equiv.) and anhydrous K_2_CO_3_ (3.5 equiv.) were refluxed in argon for 24 h. After cooling to room temperature the reaction mixture was dissolved in water and concentrated in vacuum. The crude product was extracted with dichloromethane, washed with water and brine to yield a brown solid. Purification with CC eluting with cyclohexane/ethyl acetate (9:1) gave light yellow semi-solid (**8**–**9**) (78%–86%).

*(E)-4-(4-Propoxyphenyl)3-buten-2-one* (**8**) was prepared from **7** (5.0 g, 30.5 mmol) in acetone (100 mL), 1-bromopropane (4.5 g, 36.6 mmol) and K_2_CO_3_ (14.7 g, 106.7 mmol) to give a yellow oil 5.6 g (90.0%). ^1^H-NMR δ: 7.44 (d, *J* = 8.0 Hz, 2H), 7.15 (d, *J* = 16 Hz, 1H), 6.84 (d, *J* =8.0 Hz, 2H), 6.83 (d, *J* = 16 Hz, 1H), 3.97 (t, *J* = 6.8 Hz, 2H), 2.34 (s, 3H), 1.76 (quint, *J* = 6.8 Hz, 2H), 0.94 (t, *J* = 6.8 Hz, 3H). ^13^C-NMR δ: 195.8, 163.3, 143.7, 128.6, 126.2, 116.0, 67.5, 32.6, 26.7, 24.7.

*(E)-4-(4-Pentoxyphenyl)3-buten-2-one* (**9**) was prepared from **7** (5.0 g, 30.5 mmol) in acetone (100 mL), 1-bromopentane (5.5 g, 36.6 mmol) and K_2_CO_3_ (14.7 g, 106.7 mmol) to give a yellow oil 5.8 g (88.5%). ^1^H-NMR δ: 7.45 (d, *J* = 8.0 Hz, 2H), 7.17 (d, *J* = 16 Hz, 1H), 6.86 (d, *J* =8.0 Hz, 2H), 6.85 (d, *J* = 16 Hz, 1H), 3.96 (t, *J* = 6.8 Hz, 2H), 2.37 (s, 3H), 1.80 (quint, *J* = 6.8 Hz, 2H), 1.45–1.37 (m, 4H), 0.95 (t, *J* = 6.8 Hz, 3H). ^13^C-NMR δ: 194.3, 163.5, 143.8, 128.6, 126.2, 116.2, 68.0, 32.6, 29.4, 28.2, 26.7, 24.4.

#### 3.2.5. Synthesis of (*E*)-13-(*N*-Methylpiperazinyl)-3-tridecen-2-one (**15**)

The methyl ketone bearing terminal *N*-methylpiperazine group was prepared from the corresponding (*E*)-13-bromo-3-tridecen-2-one (**14**) (1.0 equiv.), *N*-methylpiperazine (1.5 equiv.) and K_2_CO_3_ (4 equiv.) in DMF (20 mL) as previously described method for **4**. ^1^H-NMR δ: 6.91 (dt, *J* = 16.0, 6.6 Hz, 1H), 6.09 (d, *J* = 6.1 Hz, 1H), 2.65 (t, *J* = 7.6 Hz, 2H), 2.57 (s, 3H), 2.45 (m, 4H), 2.31 (m, 4H), 2.28 (s, 3H), 2.25 (q, *J* = 7.6 Hz, 2H), 1.93 (quint, *J* = 6.8 Hz, 2H), 1.49 (m, 4H), 1.28–1.21 (m, 8H). ^13^C-NMR δ: 198.6, 148.4, 132.5, 54.9, 53.0, 45.8, 35.6, 30.7, 29.6, 29.3, 29.2, 29.1, 28.8, 28.1, 26.9, 26.6.

#### 3.2.6. Synthesis of 7-Cycloheptylideneheptanoic Acid (24)

Compound **24** was obtained according to Wube *et al*. [22] from cycloheptanone (1 equiv.) in THF (100 mL), 4-(carboxybutyl)triphenylphosphonium bromide (1 equiv.) and 40% solution of sodium hexamethyldisilylamide (2 equiv.) as a light yellow oil. ^1^H-NMR δ: 11.46 (brs, 1H), 5.04 (t, *J* = 7.6 Hz, 1H), 2.35 (t, *J* = 7.2 Hz, 2H), 2.11 (m, 4H), 1.92 (q, *J* = 7.2 Hz, 2H), 1.55–1.39 (m, 10H), 1.25 (quint, *J* = 7.6 Hz, 2H). ^13^C-NMR δ: 179.7, 140.7, 126.4, 35.5, 34.6, 29.8, 29.6, 29.2, 29.1, 28.8, 27.2, 27.0, 23.4.

#### 3.2.7. Synthesis of 7-Cycloheptylidene-2-heptanone (**26**)

The methyl ketone **26** was obtained from 7-cycloheptylideneheptanoic acid (**24**, 1 equiv.) in THF (25 mL) and methyl lithium (2.5 equiv.) as a colourless oil. ^1^H-NMR δ: 5.06 (t, *J* = 7.2 Hz, 1H), 2.38 (t, *J* = 7.2 Hz, 2H), 2.14 (m, 4H), 2.07 (s, 3H), 1.93 (q, *J* = 7.2 Hz, 2H), 1.57–1.40 (m, 10H), 1.27 (quint, *J* = 7.6 Hz, 2H). ^13^C-NMR δ: 208.8, 141.2, 124.4, 43.5, 37.6, 29.8, 29.7, 29.6, 29.2, 29.2, 28.9, 27.2, 27.0, 23.4.

#### 3.2.8. Synthesis of 4-(1*H*)-Quinolones

Our 4-(1*H*)-quinolone derivatives were all prepared according to our previously reported procedure [13,14,21] by condensation of methyl ketones (1 equiv.) and derivatives of isatoic anhydrides (0.75 equiv.) at −78 °C in THF (15 mL) by the action of LDA (1.8 M in THF/heptane/benzene) (1 equiv.). Purification of the compounds was carried out using CC eluting with cyclohexane/ethyl acetate. The spectral data of compounds **18a**–**g**, **19**, **28**–**30**, **35**, **36a**–**b** were reported in our previous work [14,15,22].

*1-Methyl-2-[4′-(6′′-bromohexyl)phenyl]-4(1H)-quinolone* (**5a**) was prepared from **3b** (1.2 g, 4.2 mmol) in THF (15 mL), LDA (2.3 mL, 4.2 mmol) and *N*-methylisatoic anhydride (0.56 g, 3.15 mmol) in THF (10 mL) as a light yellow oil (61%). IR (ATR, cm^−1^): 3418, 2927, 2855, 1626, 1600, 1464, 1176, 758. ^1^H-NMR δ: 8.50 (dd, *J* = 8.0, 1.6 Hz, 1H, H-5), 7.71 (t, *J* = 8.0 Hz, 1H, H-7), 7.55 (d, *J* = 8.0 Hz, 1H, H-8), 7.42 (t, *J* = 7.6 Hz, 1H, H-6), 7.33 (d, *J* = 7.6 Hz, 2H, H-3′, 5′), 7.31 (d, *J* = 7.6 Hz, 2H, H-2′, 6′), 6.30 (s, 1H, H-3), 3.62 (s, 3H, N-CH_3_), 3.42 (t, *J* = 6.8 Hz, 2H, H-6′), 2.70 (t, *J* = 6.8 Hz, 2H, H-1′′), 1.89 (quint, *J* = 6.8 Hz, 2H, H-5′′), 1.68 (quint, *J* = 6.8 Hz, 2H, H-2′′), 1.49 (quint, *J* = 6.4 Hz, 2H H-4′′), 1.43 (quint, *J* = 6.8 Hz, 2H, H-3′′). ^13^C-NMR δ: 177.5 (C-4), 154.8 (C-2), 144.4 (C-4′), 141.9 (C-8a), 133.2 (C-1′), 132.2 (C-7), 128.7 (C-2′, 6′), 128.5 (C-3′, 5′), 126.8 (C-4a), 126.7 (C-5), 123.5 (C-6), 115.8 (C-8), 112.5 (C-3), 37.3 (N-CH_3_), 35.9 (C-1′′) 35.6 (C-6′′), 31.3 (C-2′′), 28.4 (C-4′′), 27.9 (C-3′′), 26.5 (C-5′′). ESI-MS *m/z* (rel. Int.): [M+2]^+^ 399 (100), [M]^+^ 397 (98), 341 (81).

*1-Methyl-2-[4′-(10′′-piperidinyldecyl)phenyl]-4-(1H)-quinolone* (**5b**) was prepared from **3a** (1.5 g, 4.4 mmol) in THF (15 mL), LDA (2.4 mL, 4.4 mmol) and *N*-methyl isatoic anhydride (0.58 g, 3.3 mmol) in THF (10 mL) as a light yellow semi-solid (53%). IR (ATR, cm^−1^): 3421, 2928, 2853, 1626, 1600, 1458, 1177, 758. ^1^H-NMR δ: 8.51 (dd, *J* = 8.0, 1.6 Hz, 1H, H-5), 7.71 (t, *J* = 8.0 Hz, 1H, H-7), 7.55 (d, *J* = 8.0 Hz, 1H, H-8), 7.41 (t, *J* = 7.6 Hz, 1H, H-6), 7.32 (d, *J* = 7.6 Hz, 2H, H-3′, 5′), 7.30 (d, *J* = 7.6 Hz, 2H, H-2′, 6′), 6.31 (s, 1H, H-3), 3.63 (s, 3H, N-CH_3_), 2.68 (t, *J* = 7.6 Hz, 2H, H-1′′-), 2.38 (m, 4H, N-(CH_2_)_2_-), 2.29 (t, *J* = 7.6 Hz, 2H, H-10′′), 1.67 (quint, *J* = 6.8 Hz, 2H, H-2′′), 1.59 (m, 4H, N-(CH_2_CH_2_-)_2_-), 1.48 (quint, *J* = 6.4 Hz, 2H H-9′′), 1.44 (quint, *J* = 6.8 Hz, 2H, N-CH_2_CH_2_CH_2_-), 1.42 (quint, *J* = 6.8 Hz, 2H, H-8′′), 1.37–1.26 (m, 10H, H-3′′-7′′). ^13^C-NMR δ: 177.6 (C-4), 154.8 (C-2), 144.5 (C-4’), 141.7 (C-8a), 133.2 (C-1′), 132.1 (C-7), 128.7 (C-2′, 6′), 128.5 (C-3′, 5′), 126.8 (C-4a), 126.7 (C-5), 123.5 (C-6), 115.9 (C-8), 112.7 (C-3), 59.6 (C-10′′), 54.6 (-N-(CH_2_)_2_-), 37.3 (N-CH_3_), 35.8 (C-1′′), 31.3 (C-2′′), 29.6, 29.4, 29.3, 29.1, 28.1, 27.7, 26.8 (C-9′′), 25.9 (-N-(CH_2_-CH_2_)_2_-), 24.4. ESI-MS *m/z* (rel. Int.): [M+H]^+^ 460 (60), 230 (100).

*1-(2-Bromoethyl)-2-[(1′E)-(4′′-propoxyphenyl)ethenyl]-4-(1H)-quinolone* (**10a**) was prepared from **8** (1.0 g, 4.9 mmol) in THF (15 mL), LDA (2.7 mL, 4.9 mmol) and *N*-(1-bromoethyl) isatoic anhydride (0.99 g, 3.7 mmol) in THF (15 mL) as a colourless semisolid (59%). IR (ATR, cm^−1^): 3422, 2927, 2853, 1627, 1600, 1472, 1460, 759. ^1^H-NMR δ: 8.46 (d, *J* = 8.0 Hz, 1H, H-5), 7.69 (t, *J* = 8.0 Hz, 1H, H-7), 7.47 (d, *J* = 7.6 Hz, 2H, H-2′′, 6′′), 7.44 (d, *J* = 8.0 Hz, 1H, H-8), 7.38 (t, *J* = 8.0 Hz, 1H, H-6), 7.16 (d, *J* = 16.0 Hz, 1H, H 2′), 6.95 (d, *J* = 16.0 Hz, 1H, H-1′), 6.94 (d, *J* = 7.6 Hz, 2H, H-3′, 5′), 6.52 (s, 1H, H-3), 4.60 (t, 2H, N-CH_2_-), 3.97 (t, *J* = 6.8 Hz, 2H, O-CH_2_-), 3.65 (t, *J* = 6.8 Hz, 2H, N-CH_2_-CH_2_-Br), 1.83 (m, 2H, O-CH_2_-CH_2_-), 1.05 (t, *J* = 7.2 Hz, 3H, O-CH_2_-CH_2_-CH_3_). ^13^C-NMR δ: 177.8 (C-4), 160.5 (C-4′′), 151.6 (C-2), 140.3 (C-8a), 138.8 (C-2′), 132.6 (C-7), 128.9 (C-2′, 6′), 127.7 (C-1′′), 127.1 (C-5), 127.0 (C-4a), 123.7 (C-6), 117.7 (C-1′), 114.9 (C-3′′, 5′′), 114.7 (C-8), 109.7 (C-3), 69.6 (O-CH_2_-), 48.3 (-N-CH_2_-), 26.4 (N-CH_2_-CH_2_-Br), 22.5 (O-CH_2_-CH_2_-), 10.5 (O-CH_2_-CH_2_-CH_3_). ESI-MS *m/z* (rel. Int.): [M+H]^+^ 414 (100), 412 (78).

*6-Chloro-1-methyl-2-[(1′E)-(4′′-propoxyphenyl)ethenyl]-4-(1H)-quinolone* (**10b**) was prepared from **8** (1.0 g, 4.9 mmol) in THF (15 mL), LDA (2.7 mL, 4.9 mmol) and 6-chloro-*N*-methyl isatoic anhydride (0.78 g, 3.7 mmol) in THF (10 mL) as a light yellow oil (54%). IR (ATR, cm^−1^): 3420, 2929, 2857, 1626, 1600, 1466, 1175, 760. ^1^H-NMR δ: 8.32 (d, *J* = 8.0 Hz, 1H, H-5), 7.51 (d, *J* = 8.0 Hz, 1H, H-7), 7.42 (d, *J* = 7.6 Hz, 2H, H-2′′, 6′′), 7.36 (d, *J* = 8.0 Hz, 1H, H-8), 7.06 (d, *J* = 16.0 Hz, 1H, H 2′), 6.91 (d, *J* = 7.6 Hz, 2H, H-3′, 5′), 6.84 (d, *J* = 16.0 Hz, 1H, H-1′), 6.41 (s, 1H, H-3), 3.95 (t, *J* = 6.8 Hz, 2H, O-CH_2_-), 3.74 (s, 3H,N-CH_3_), 1.82 (m, 2H, O-CH_2_-CH_2_-), 1.05 (t, *J* = 7.2 Hz, 3H, O-CH_2_-CH_2_-CH_3_). ^13^C-NMR δ: 176.4 (C-4), 160.4 (C-4′′), 152.4 (C-2), 139.9 (C-8a), 137.9 (C-2′), 129.5 (C-1′′), 128.8 (C-2′, 6′), 127.7 (C-5), 127.6 (C-4a), 123.7 (C-7), 111.0 (C-6), 117.3 (C-1′), 114.9 (C-3′′, 5′′), 118.5 (C-8), 109.2 (C-3), 69.6 (O-CH_2_-), 35.7 (N-CH_3_), 22.4 (O-CH_2_-CH_2_-), 10.4 (O-CH_2_-CH_2_-CH_3_). ESI-MS *m/z* (rel. Int.): [M+H]^+^ 356 (40), 354 (100), 312 [M+H−C_3_H_7_]^+^.

*1-(2-Bromoethyl)-2--[(1′E)-(4′′-propoxyphenyl)ethenyl]-4-(1H)-quinolone* (**11**) was prepared from **9** (1.1 g, 4.7 mmol) in THF (15 mL), LDA (2.6 mL, 4.7 mmol) and *N*-(1-bromoethyl) isatoic anhydride (0.95 g, 3.5 mmol) in THF (15 mL) as a colourless semisolid (51%). IR (ATR, cm^−1^): 3430, 2926, 2853, 1625, 1600, 1482, 1464, 1198, 757. ^1^H-NMR δ: 8.44 (d, *J* = 8.0 Hz, 1H, H-5), 7.67 (t, *J* = 8.0 Hz, 1H, H-7), 7.45 (d, *J* = 7.6 Hz, 2H, H-2′′, 6′′), 7.42 (d, *J* = 8.0 Hz, 1H, H-8), 7.36 (t, *J* = 8.0 Hz, 1H, H-6), 7.15 (d, *J* = 16.0 Hz, 1H, H 2′), 6.93 (d, *J* = 16.0 Hz, 1H, H-1′), 6.93 (d, *J* = 7.6 Hz, 2H, H-3′, 5′), 6.49 (s, 1H, H-3), 4.59 (t, 2H, N-CH_2_-), 3.40 (t, *J* = 6.8 Hz, 2H, O-CH_2_-), 3.65 (t, *J* = 6.8 Hz, 2H,N-CH_2_-CH_2_-Br), 1.79 (m, 2H, O-CH_2_-CH_2_-), 1.44 (m, 4H, O-CH_2_-CH_2_-CH_2_-CH_2_-), 0.97 (t, *J* = 7.2 Hz, 3H, O-CH_2_-CH_2_-CH_2_-CH_2_-CH_3_). ^13^C-NMR δ: 177.7 (C-4), 160.4 (C-4′′), 151.5 (C-2), 140.2 (C-8a), 138.8 (C-2′), 132.5 (C-7), 128.9 (C-2′, 6′), 127.6 (C-1′′), 127.0 (C-5), 126.9 (C-4a), 123.6 (C-6), 117.7 (C-1′), 114.9 (C-3′′, 5′′), 114.7 (C-8), 109.7 (C-3), 68.1 (O-CH_2_-), 48.3 (-N-CH_2_-), 28.8 (O-CH_2_-CH_2_-), 28.1 (O-CH_2_-CH_2_-CH_2_-), 26.5 (N-CH_2_-CH_2_-Br), 22.4 (O-CH_2_-CH_2_-CH_2_-CH_2_-), 14.0 (O-CH_2_-CH_2_-CH_2_-CH_2_-CH_3_). ESI-MS *m/z* (rel. Int.): [M+H]^+^ 442 (100), 440, 361 [M+H−Br]^+^.

*1-Methyl-2-[(1′E)-11′(N-methylpiperazinyl)undecenyl]-4-(1H)-quinolone* (**20**) was prepared from **16** (1.0 g, 3.4 mmol) in THF (15 mL), LDA (1.9 mL, 3.4 mmol) and *N*-methyl isatoic anhydride (0.45 g, 5.55 mmol) in THF (10 mL) as a colourless oil (59%). IR (ATR, cm^−1^): 3420, 2926, 2853, 1625, 1602, 1465, 1443, 1176, 759. ^1^H-NMR δ: 8.43 (dd, *J* = 8.0, 1.6 Hz, 1H, H-5), 7.66 (t, *J* = 8.0 Hz, 1H, H-7), 7.51 (d, *J* = 8.0 Hz, 1H, H-8), 7.37 (t, *J* = 7.6 Hz, 1H, H-6), 6.43 (d, *J* = 16.0 Hz, 1H, H-1′), 6.37 (s, 1H, H-3), 6.32 (d, *J* = 16.0 Hz,12H, H-2′), 3.74 (s, 3H, N-CH_3_), 2.54 (t, *J* = 6.8 Hz, 4H, N-(CH_2_-CH_2_-)_2_), 2.52 (t, *J* = 6.8 Hz, 4H, N-(CH_2_-CH_2_-)_2_), 2.33 (t, *J* = 7.2 Hz, H-11′), 2.29 (t, *J* = 7.2 Hz, 2H, H-3′), 2.24 (s, 3H, -N-CH_3_), 1.52 (quint, *J* = 6.8 Hz, 2H, H-10′), 1.49 (quint, *J* = 6.8 Hz, 2H, H-4′), 1.41 (quint, *J* = 6.8 Hz, 2H, H-9′), 1.33–1.23 (m, 8H, H-5′-8′). ^13^C-NMR δ: 177.9 (C-4), 152.3 (C-2), 141.5 (C-2′), 141.3 (C-8a), 132.1 (C-7), 126.8 (C-4a), 126.6 (C-5), 123.3 (C-6), 123.1 C-1′), 115.4 (C-8), 109.0 (C-3), 58.8 (C-11′), 55.1 (N-(CH_2_-)_2_), 53.1 (N-(CH_2_-CH_2_-)_2_), 46.0 (N-CH_3_), 35.4 (N-CH_3_), 33.1 (C-3′), 29.5, 29.4, 29.3, 29.1, 29.0, 28.6 (C-4′), 26.9 (C-10′). ESI-MS *m/z* (rel. Int.): [M]^+^ 410 (60), 206 (100).

*1-(2-Bromoethyl)-2-[(1′E)-2′-(2′′,6′′,6′′-trimethyl-1′′-cyclohexen-1′′-yl)ethenyl]-4-(1H)-quinol-one* (**21**) was prepared from β-ionone (1.5 g, 7.8 mmol) in THF (15 mL), LDA (4.3 mL, 7.8 mmol) and *N*-(1-bromoethyl) isatoic anhydride (1.6 g, 5.9 mmol) in THF (20 mL) as a light yellow oil (62%). IR (ATR, cm^−1^): 3422, 2928, 2849, 1624, 1601, 1464, 1173, 756. ^1^H-NMR δ: 8.47 (dd, *J* = 8.0, 1.6 Hz, 1H, H-5), 7.70 (t, *J* = 8.0 Hz, 1H, H-7), 7.45 (d, *J* = 8.0 Hz, 1H, H-8), 7.41 (t, *J* = 7.6 Hz, 1H, H-6), 6.85 (d, *J* = 16.0 Hz, 1H, H-2′), 6.48 (s, H-3), 6.41 (d, *J* = 16.0 Hz, 1H), H-1′), 4.56 (t, *J* = 7.2 Hz, 2H, N-CH_2_-), 3.59 (t, *J* = 7.2 Hz, 2H, N-CH_2_-CH_2_-Br), 2.09 (t, *J* = 6.4 Hz, 2H, H-3′′), 1.82 (s, 3H, 2′′-CH_3_), 1.67 (m, 2H, H-4′′), 1.53 (t, *J* = 6.4 Hz, 2H, H-5′′), 1.10 (s, 6H, 6′′-(CH_3_)_2_). ^13^C-NMR δ: 178.0 (C-4), 151.9 (C-2), 140.2 (C-8a), 139.0 (C-2′), 136.7 (C-1′′), 132.6 (C-7), 132.5 (C-2′′), 127.2 (C-5), 126.9 (C-4a), 125.2 (C-1′), 123.7 (C-6), 114.6 (C-8), 110.1 (C-3), 48.4 (N-CH_2_-), 39.3 (C-5′′), 34.3 (C-6′′), 33.0 (C-3′′), 28.9 (6′′-(CH_3_)_2_), 26.1 (N-CH_2_-CH_2_-Br), 21.7 (2′′-CH_3_), 19.0 (C-4′′). ESI-MS *m/z* (rel. Int.): [M+H]^+^ 402 (100), 400, [M+H−Br]^+^ 321.

*1-Methyl-2-[(5′E)-cycloheptylidenylpentynyl]-4-(1H)-quinolone* (**31**) was prepared from **27** (1.0 g, 4.8 mmol) in THF (15 mL), LDA (2.7 mL, 4.8 mmol) and *N*-methyl isatoic anhydride (0.64 g, 3.6 mmol) in THF (10 mL) as a light yellow solid (63%). IR (ATR, cm^−1^): 3425, 2923, 2852, 1619, 1598, 1462, 1174, 759. ^1^H-NMR δ: 8.42 (dd, *J* = 8.0, 1.6 Hz, 1H, H-5), 7.65 (t, *J* = 8.0 Hz, 1H, H-7), 7.49 (d, *J* = 8.0 Hz, 1H, H-8), 7.36 (t, *J* = 7.6 Hz, 1H, H-6), 6.23 (s, 1H), H-3), 5.10 (t, *J* = 6.8 Hz, 1H, H5′), 3.72 (s, 3H, N-CH_3_), 2.69 (t, *J* = 7.6 Hz, 2H, H-1′), 2.20 (m, 4H, H-2′′, 7′′), 2.02 (q, *J* = 6.8 Hz, 2H, H-4′), 1.67 (quint, *J* = 6.8 Hz, 2H, H-2′), 1.54 (m, 4H, H-3′′, 6′′), 1.48 (m, 4H, H-4′′, 5′′), 1.46 (quint, *J* = 6.4 Hz, 2H, H-3′). ^13^C-NMR δ: 177.6 (C-4), 154.9 (C-2), 141.9 (C-8a), 141.8 (C-1′′), 132.1 (C-7), 126.4 (C-5), 126.2 (C-4a), 124.0 (C-5′), 123.3 (C-6), 115.3 (C-8), 110.8 (C-3), 34.6 (C-1′), 34.1 (N-CH_3_), 30.0 (C-2′′, 7′′), 29.8 (C-4′′, 5′′), 29.2 (C-3′′, 6′′), 29.0 (C-3′′), 28.0 (C-2′), 27.0 (C-4′). ESI-MS *m/z* (rel. Int.): [M−CH_3_]^+^ 324 (100), 186, 173.

### 3.3. Biological Evaluation

#### 3.3.1. Antimalarial Assay

Antimalarial activity *in vitro* was determined by means of the microculture radioisotope technique which makes use of ^3^H-hypoxanthine as an indicator of parasite viability [23]. In this assay the drug sensitive NF54 strain was employed. Parasite cultures supplemented with HEPES (5.94 g/L), NaHCO_3_ (2.1 g/L), neomycin (100 U/mL), Albumax^®^ (5 g/L) and washed human red cells A^+^ at 2.5% haematocrit (0.3% parasitaemia) were incubated in RPMI 1640 medium without hypoxanthine and placed in the 96-well plates. Solutions of 4-(1*H*)-quinolone derivatives and the positive control chloroquine were dissolved in DMSO at 10 mg/mL and added to the first well followed by serial dilution to afford test concentrations ranging from 100 to 0.002 mg/L. After 48 h incubation at 37 °C, 4% CO_2_, 3% O_2_, 93% N_2_, ^3^H-hypoxyanthine was added (0.5 µC/well) and then the plates were incubated further for 24 h under the same condition. The plates were freeze/thawed rapidly, harvested on glass fibre filters and dried. Radioactive hypoxanthine uptake was measured by scintillation counter. IC_50_ values were calculated from sigmoidal inhibition curves by linear regression [24] using Microsoft Excel. Assays were carried out two times in triplicate.

#### 3.3.2. Antitrypanosomal Assay

*In vitro* antitrypanosomal assay was carried out against the bloodstream form of *T. b. rhodesiense* STIB 900 strain using the Alamar Blue assay. The STIB 900 strain was suspended in fresh medium containing 1% MEM powder medium, MEM non-essential amino acids (100x) supplemented with 25 mM HEPES, additional glucose (1 g/L), 0.2 mM 2-mercaptoethanol, 1.0 mM sodium pyruvate and 15% heat inactivated horse serum [17]. 50 µL (4 × 10^3^ trypanosomes/mL) of this suspension was seeded in each well of the 96-well microtiter plate which contained test compounds and the positive control melarsoprol which were dissolved in DMSO and serially diluted to concentrations ranging from 90 to 0.123 µg/L. The plates were incubated at 37 °C under 5% CO_2_ atmosphere for 70 h. Alamar Blue solution (10 µL) was added and incubation was continued for further 2–4 h [25]. Plates were read with a Spectramax Gemini XS (Molecular Devices Cooperation, Sunnyvale, CA, USA) at EX/EM (536/588 nm). The IC_50_ values were calculated by linear regression from the sigmoidal dose inhibition curves using SoftmaxPro software. Tests were carried out two times in triplicate.

#### 3.3.3. Cytotoxicity Assay

96-well plates were seeded with 100 µL of RPMI 1640 medium supplemented with 1% l-glutamine and 10% fetal bovine serum, and rat skeletal muscle cell (L-6) (4 × 10^3^/mL). The 4-(1*H*)-quinolone derivatives and the positive control podophyllotoxin were dissolved in DMSO and with a serial dilution ranging from 100 to 0.002 µg/L were added into the wells in triplicate. The plates were incubated for 70 h and assessed microscopically for cell growth. Alamar Blue solution (10 µL) was then added to each well and incubation continued for 2 h before reading with a Spectramax Gemini XS at EX/EM 536/588. The IC_50_ values were calculated by linear regression from the sigmoidal dose inhibition curves using SoftmaxPro software. Tests were carried out two times in triplicate.

## 4. Conclusions

To elucidate the structural requirement for antiprotozoal properties of 1,2-substituted 4-(1*H*)-quinolones, a diverse array of compounds were synthesized and tested against *P. falciparum* and *T.b. rhodesiense*. Results of our study revealed that phenylalkyl substituents at position 2 largely dictated antimalarial potencies and the introduction of bromine at the end of the aliphatic side chain improved the antimalarial activities. Moreover, lipophilicity and unsaturation are detrimental for the inhibitory effect. Compound **5a**, in particular, was found to be the most potent, with an IC_50_ value of 90 nM and selectivity index of 73. Although, compound **10b** showed a ten-fold less potency compared to **5a**, its selectivity index was found to be superior. Compounds **5a** and **10b** could therefore serve as important antimalarial lead for further studies.
